# The complete chloroplast genome of *Epimedium brevicornu* (Berberidaceae), a traditional Chinese medicinal herb

**DOI:** 10.1080/23802359.2020.1718027

**Published:** 2020-01-27

**Authors:** Yu Yao, Xiang Liu, Qianru Yang, Yanjiao Luo, Cheng Zhang, Chaoqun Xu, Fengmei Suo, Guoan Shen, Baolin Guo

**Affiliations:** a Institute of Medicinal Plant Development, Chinese Academy of Medical Science, Peking Union Medical College, Beijing, China;; b Chongqing Academy of Chinese Materia Medica, Chongqing, China;; c College of Pharmacy, Shanxi Medical University, Taiyuan, China

**Keywords:** Chloroplast genome, *Epimedium brevicornu*, Berberidaceae

## Abstract

Epimedii Folium has been used as a common traditional Chinese medicine for more than 2000 years in China. In this study, we assembled the complete chloroplast (cp) genome of *Epimedium brevicornu*. The whole cp genome of *E. brevicornu* is 158,658 bp in length, comprising a pair of inverted repeat (IR) regions (27,699 bp) separated by a large single copy (LSC) region (86,558 bp) and a small single copy (SSC) region (16,702bp). The *E. brevicornu* cp genome contains 129 genes, of which 84 protein-coding genes, 37 tRNA genes, and eight rRNA genes. Phylogenetic analysis shows that *E*. *brevicornu* is closely clustered with *E. wushanense*, *E. lishihchenii*, and *E. sagittatum*. The published *E. brevicornu* chloroplast genome will provide useful information for the phylogenetic and evolutionary study on *Epimedium* family of Berberidaceae.

Epimedii Folium, also known as Yinyanghuo, has been widely used as Traditional Chinese Medicines (TCM) for more than two thousand years in China. The leaves of some plant species in the *Epimedium* family have beneficial effects on human beings and animals as tonic, aphrodisiac and antirheumatics in curing sexual dysfunction and osteoporosis (Ma et al. 2011). *Epimedium brevicornu* Maxim. and other three species of the genus *Epimedium* L. are used as medicinal materials of Epimedii Folium according to Chinese Pharmacopoeia Commission’s (2015) suggestion. *Epimedium* family is taxonomically and phylogenetically regarded as one of the most challengingly difficult taxa in plant kingdom (Zhang et al. 2016). Since Epimedii Folium were mainly from wild resources in the market, the species confusion of *Epimedium* resulted in uncontrollable quality and unstable curative effects of medicinal materials (De Smet et al. 2012). The chloroplast genomes are conservative, thus it is more efficient to distinguish closely related plants at the species and population levels by using the chloroplast genome than chromosome genomic DNA regions (Li et al. 2015). Therefore, we reported the complete chloroplast genome sequence of *E. brevicornu* in the present study.

The total genomic DNA was extracted from the fresh leaves of *E. brevicornu* with a modified CTAB method (Doyle and Doyle 1987). The voucher sample (18,042) were cultivated in the Lixian County of Gansu Province, China (N34°06′, E105°32′) and deposited at the Herbarium of the Institute of Medicinal Plant (IMPLAD), Beijing, China. The 500 bp shotgun library construction was prepared from total DNA. The sequencing was performed using Illumina Novaseq PE150 platform. The filtered reads were assembled using the program GetOrganelle (Jin et al. 2018) with the reference chloroplast genome of *E. acuminatum* (GenBank accession number: NC_029941), the chloroplast genome was annotated through the online program CPGAVAS2 (Shi et al. 2019) and GeSeq (Tillich et al. 2017), followed by manual correction. The annotated chloroplast genome sequence has been deposited into GenBank (Accession number MN803415). The phylogenetic tree was generated based on the shared protein-coding genes among *E. Brevicornu* and other 20 species retrieved from the NCBI GenBank database. MAFFT v7 (Katoh et al. 2017) was used to align the sequences, and a maximum likelihood tree was generated by using RAxML v8.2.10 (Stamatakis 2014), with *Sinofranchetia chinensis* as the outgroup.

The complete chloroplast genome size of *E. brevicornu* is 158,658 bp in length, containing a large single-copy (LSC) region of 86,559 bp, a small single-copy (SSC) region of 16,703 bp, and two inverted repeat (IRa and IRb) regions of 27,698 bp. The total GC content of complete chloroplast genome, LSC, SSC, and IR regions is 38.85%, 37.29%, 33.01%, and 43.06%, respectively. In total, 129 genes were annotated, including 84 protein-coding genes, 37 tRNA genes, and eight rRNA genes. One *trnQ-UUG* gene is duplicated in the LSC regions. Six protein-coding genes (*rpl2*, *rp123*, *ndhB*, *rps7*, *rps19*, and *ycf2*), seven tRNA genes (*trnI-CAU*, *trnL-CAA*, *trnV-GAC*, *trnI-GAU*, *trnA-UGC*, *trnR-ACG*, and *trnN-GUU*), and four rRNA genes (*rrn16*, *rrn23*, *rrn4.5*, and *rrn5*) are duplicated in the IR regions. In these genes, 15 genes (six tRNA genes and nine protein-coding genes) contain one intron, and three genes (*ycf3*, *clpP*, and *rps12*) contain a couple of introns. The 5′ end and 3′ end of *rps12* gene is duplicated in the LSC regions and IR regions, respectively. The *rps12* gene is trans-spliced.

Phylogenetic analysis shows that *E*. *brevicornu*is is closely clustered with *E. wushanense*, *E. lishihchenii*, and *E. sagittatum* (Figure 1). The published *E. brevicornu* chloroplast genome will provide useful information for the phylogenetic and evolutionary study on *Epimedium* family of Berberidaceae.

**Figure 1.**
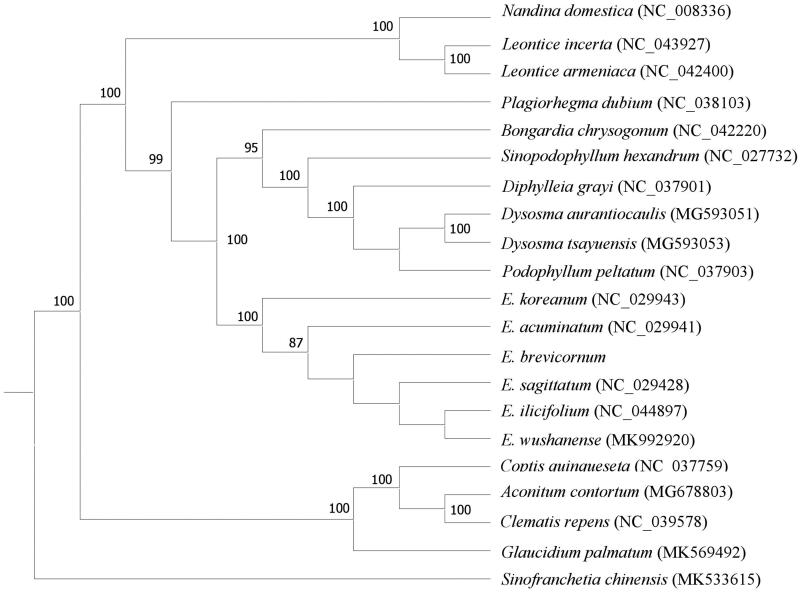
ML phylogenetic tree was constructed based on the shared protein-coding genes shared between the 20 species.
